# Public concerns over presumed metal and radionuclide pollution: testing a possible link to ovine hepatic melanosis in South Greenland

**DOI:** 10.1007/s10661-025-14945-z

**Published:** 2026-01-22

**Authors:** Violeta Hansen, Ole Lerberg Nielsen, Anders Mosbech, Sandra Drewes Fabricius, Christian Sonne, Jens Søndergaard, Daniel Spelling Clausen, Kasper Lambert Johansen, Floris van Beest, Páll Skúli Leifsson, Heidi Larsen Enemark

**Affiliations:** 1 Institute of Clinical Sciences, Medical Radiation Sciences, Sahlgrenska Academy, University of Gothenburg, Gula Stråket 2B, Göteborg, SU 41345 Sweden; 2 Department of Fisheries and Hunting, Government of Greenland, Veterinary and Food Authority in Greenland, P.O. Box 269, Imaneq 1 A 701, Nuuk, Greenland; 3 Department of Ecoscience, Aarhus University, Frederiksborgvej 399, Roskilde, 4000 Denmark; 4 Department of Veterinary and Animal Sciences, University of Copenhagen, Grønnegårdsvej 15, Frederiksberg C, 1870 Denmark; 5 Department of Animal and Veterinary Sciences, Aarhus University, Blichers Allé 20, Tjele, 8830 Denmark

**Keywords:** Arctic, Hepatic lipofuscinosis, Black livers, Heavy metals, Naturally occurring radionuclides, Prevalence

## Abstract

In South Greenland, public concerns have linked “black livers” in sheep to presumed environmental pollution from the Kvanefjeld mining exploration project. Elevated concentrations of metals and radionuclides in the area reflect natural geochemistry and weathering processes, not pollution from exploration activities. However, in response, we conducted a preliminary case–control study to diagnose the condition, investigate links between liver discoloration and environmental concentrations of metals and radionuclides, and to estimate the annual effective dose to the public from polonium-210 (^210^Po) ingestion via sheep and lamb liver consumption. We also evaluated the prevalence of acquired melanosis, grazing and herd effects, and their association with carcass quality and disease markers. Histological analysis confirmed acquired melanosis in affected liver samples. No statistically significant differences in concentrations of chemical elements or radionuclides (including ^21^^0^Po and ^21^^0^Pb) were found between case and control groups, across age groups, grazing areas, or farms. Except for the most exposed children (10% percentile), the estimated annual effective dose to adults and children from ^210^Po ingestion in black and healthy livers remained below the world average of 120 µSv due to the ingestion of naturally occurring radionuclides. The overall prevalence of ovine acquired melanosis based on a large sample size was 10.59%, with herd prevalence ranging from 0% to 51.79%. Age-specific prevalence was 10.38% in lambs and 13.40% in adult sheep. Acquired melanosis was negatively correlated with fat and muscle content in carcasses. The consumption of sheep livers from South Greenland does not appear to pose a toxicological or radiological risk.

**Graphical Abstract:**

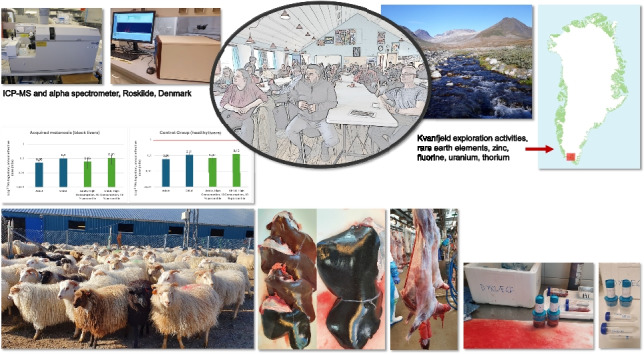

**Supplementary information:**

The online version contains supplementary material available at 10.1007/s10661-025-14945-z.

## Introduction

Acquired melanosis syn. hepatic lipofuscinosis is a condition that affects sheep (*Ovis aries*) and, to a lesser extent, cattle (*Bos taurus*). Acquired melanosis is characterised by a uniform grey to black discoloration of the liver—occasionally accompanied by similar discoloration of hepatic lymph nodes, lungs, and renal cortex (Cullen & Stalker, 2015). Few studies have addressed hepatic acquired melanosis globally, likely due to the apparent absence of clinical signs associated with the condition. Most reports are based exclusively on macroscopic observations at slaughter in otherwise healthy animals (McCrea, 1970). Documented cases originate from Australia (Winter, 1961), Norway (Nordstoga, 1990), and the Falkland Islands (McCrea, 1970); however, to our knowledge, no true prevalence studies have been conducted in Greenland, Scandinavia, or elsewhere in the world. Lipofuscin pigment was identified by Winter (1961) and Nordstoga (1990), and the condition was subsequently proposed to be hepatic lipofuscinosis rather than acquired melanosis (Cullen & Stalker, 2015; Nordstoga, 1990). The condition has also been (1) named black liver, acquired visceral melanosis, or black organ disease, (2) claimed to affect goats, and (3) declared to occur in India and Iran (Meat and Livestock Australia homepage, 2018; Australian Livestock Export Corporation Limited, 2023).

An unequivocal identification of the pigment causing the discoloration seen in acquired melanosis is still lacking. Current hypotheses suggest the involvement of melanin, lipofuscin, or a combination of both (Meat and Livestock Australia homepage, 2018). Likewise, the aetiology and pathogenesis of the condition remain unknown. Nordstoga (1990) proposed a potential association with specific soil types and/or pasture vegetation, noting that many affected animals had grazed on mountain pastures during the summer months. However, this hypothesis is based exclusively on observational evidence and post-mortem findings, with no supporting empirical data. In Australia, although direct experimental confirmation is lacking, the circumstantial evidence has been considered sufficiently strong to support a nutritional origin of the pigmentation observed in sheep and other animals grazing in areas with *mulga* vegetation. Notably, similar pigmentation has not been reported outside regions where *mulga* vegetation is present (Winter, 1961). The pigment does not appear to be taken orally from external sources but is instead produced endogenously in the liver (Winter, 1961).

The formation and pathological significance of lipofuscin have been extensively reviewed (Baldersberg et al., 2024; Moreno-Garcia et al., 2018; Różanowska, 2023). Traditionally, lipofuscin has been regarded as an inert “wear-and-tear” pigment that accumulates progressively within cells as a consequence of ageing. Its deposition occurs predominantly in neurons and cardiomyocytes, i.e. post-mitotic cell populations where dilution through cell division is not possible. Lipofuscin originates from incomplete degradation of intracellular components, including damaged organelles and macromolecules, reflecting impaired cellular clearance mechanisms. This dysfunction is linked to cellular senescence, genetic predispositions, and oxidative stress. The composition of lipofuscin varies by tissue type but generally consists of a heterogeneous mixture of proteins, lipids, trace carbohydrates, and metal cations such as Fe, Cu, and Zn. These metals confer pro-oxidant properties, enabling lipofuscin itself to contribute to oxidative damage and further cellular decline.

In Greenland, commercial sheep farming is only possible in the coastal, southwestern part of the country, which has a sub-to-low Arctic climate (Austrheim et al., 2008). From late October to May, the animals are stabled and fed locally produced hay and imported supplementary concentrate. The lambing season is in May–June, and all sheep graze extensive mountain pastures from June to October. The slaughter season extends from early September to early November, and all animals are slaughtered at the Neqi A/S abattoir in Narsaq town, where they are subject to veterinary post-mortem inspection. Systematic recording of discoloured livers (acquired melanosis) was implemented in 2021, but according to locals, including personnel at Neqi A/S, discoloured livers have been present for at least 20 years. The objectivity of residents’ accounts may involve some degree of uncertainty.

In comparison with other regions in Greenland, naturally elevated background concentrations of fluorine (F), rare earth elements (REE), zinc (Zn), uranium (U), thorium (Th), and polonium (Po) in soil, river sediments, vegetation, fjord, freshwater, and drinking water have been reported in the environment at the Kvanefjeld (Kuannersuit in Greenlandic) deposit, located near Narsaq town in South Greenland, where some farmers graze their sheep (Pilegaard, 1990; EIA, 2020; Hansen et al., 2019a, b, 2020, 2022). Based on previous assessments, the currently elevated concentrations of F, REE, Zn, U, Th, and Po in the area appear to be natural, resulting from local geochemistry and weathering processes rather than exploration-related activities (Pilegaard, 1990; EIA, 2020; Hansen et al., 2019a, b, 2020, 2022). However, future extraction activities could potentially increase these concentrations and pose a pollution risk.

**Fig. 1:**
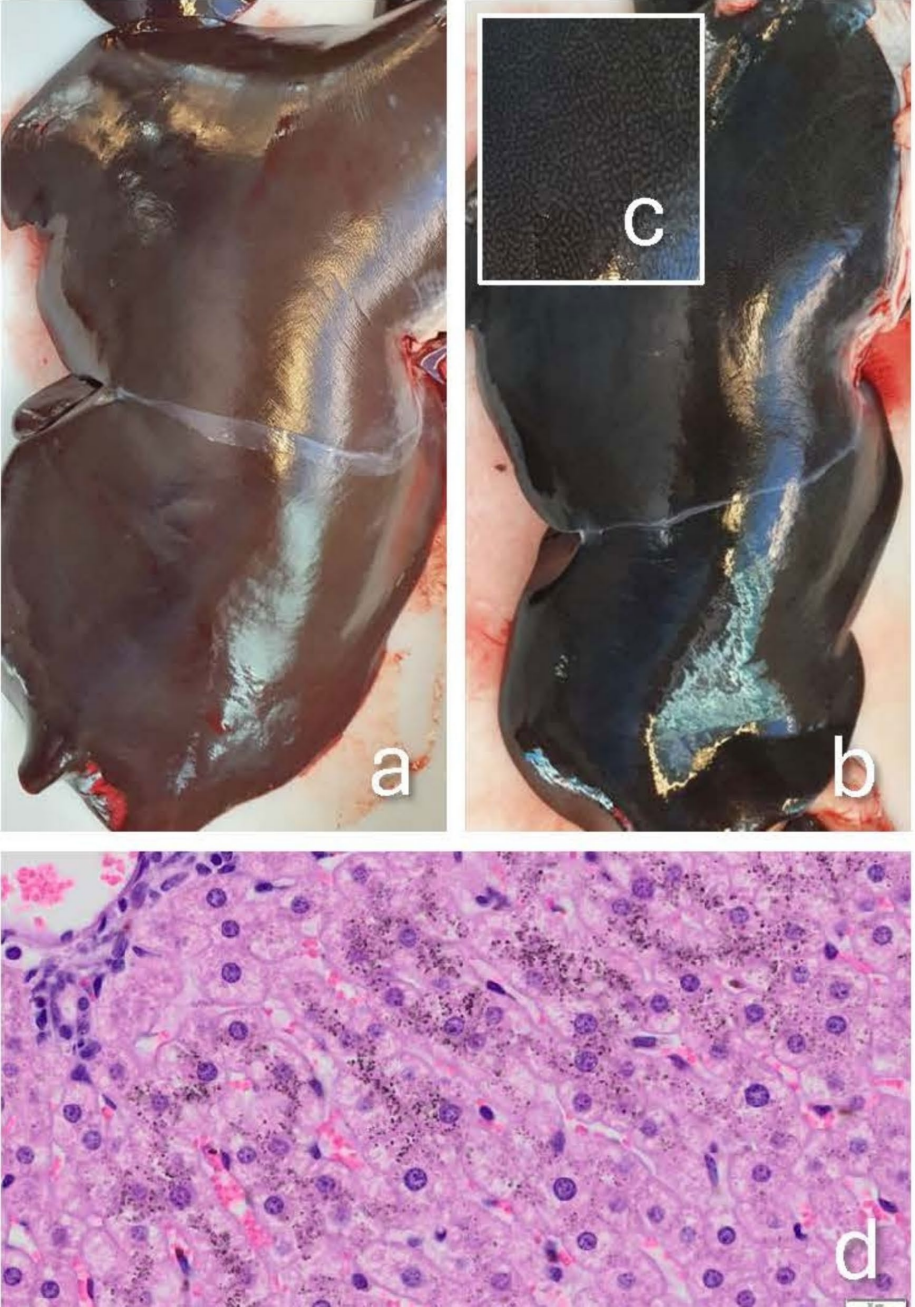
Morphology of ovine livers with and without acquired melanosis. **a** Normal liver (= control). **b** Liver with acquired melanosis (= case); **c** The black pigmentation has a reticular pattern, projecting the basic and repeated lobular structure of the liver, i.e. it is not homogenously distributed throughout the liver tissue. **d** Histology of ovine liver (CA11) with acquired melanosis. The black dots are responsible for the black discoloration and are graded histologically as severe (grade 5). Haematoxylin and eosin staining; × 40 magnification; bar 20 µm

Public concerns and ongoing debates in South Greenland have associated the occurrence of “black livers” (Fig. 1) in sheep with environmental pollution from Kvanfjeld exploration activities contributing to resistance against the mining project. To address this, we conducted a preliminary case–control study in 2021 with the following objectives: (1) determine whether black discoloration of sheep livers corresponds to the diagnosis of acquired melanosis, (2) uncover possible correlations between black discoloration of the livers and concentrations of metals, REE, ^210^Po, and ^210^Pb in affected (case) versus unaffected (control) livers, (3) calculate the annual absorbed dose due to ^210^Po in the liver samples, and (4) estimate the annual radiation effective dose to average Greenlandic adults, children, and the most exposed individuals (defined as the top 10% of liver consumers), based on consumption of sheep livers with and without acquired melanosis.

Additionally, we included data on the total number of sheep slaughtered in South Greenland in 2021 to support a prevalence study aimed at (5) assessing the prevalence of acquired melanosis and evaluating the influence of grazing area and herd, and (6) exploring correlations between acquired melanosis and carcass quality, including fat and muscle content, as well as other disease markers.

## Materials and methods

### Case–control study

**Fig. 2:**
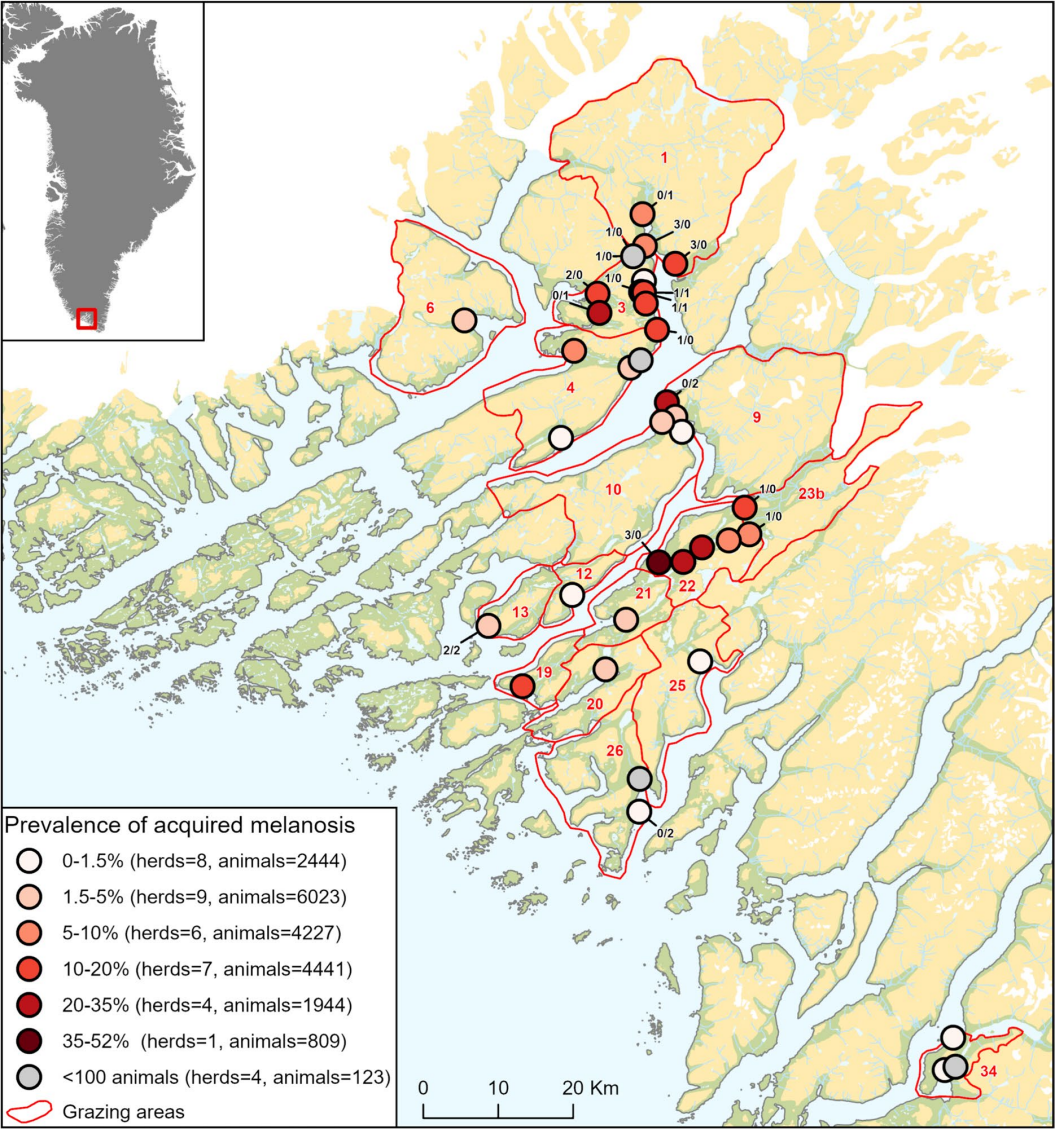
The prevalence of acquired melanosis in slaughtered sheep and lambs in South Greenland, 2021. Each dot represents a farm (a herd), colour-coded according to the prevalence of acquired melanosis (in %), except for farms with fewer than 100 slaughtered animals, which are shown as grey dots due to low sample size. The red polygons are the grazing areas used by the farms (designated with a grazing area ID number in red). The small, black labels on the farms indicate the number of case/control samples of livers used in the case–control study

During post-mortem inspections in 2021, a limited number of livers and blood samples were randomly collected from 17 different herds. This pilot study included livers with macroscopically visible black discoloration (cases, *n* = 21) and normal-appearing livers (controls, *n* = 10) (Fig. 1). Liver classification (case vs. control) was performed independently by two experienced veterinarians based on visual assessment, as no standardised diagnostic criteria currently exist. The diagnosis of acquired melanosis was subsequently confirmed through histological examination (see section “Histology”). The geographical origin of these samples is illustrated in Fig. 2. Animal identity, herd identity, age (lamb or sheep), slaughter date, and liver pigmentation detected post-mortem and histologically are presented in Table 1. Liver samples for histology were fixed in 4% neutral buffered formaldehyde (10% formalin) and shipped to the University of Copenhagen, Department of Veterinary and Animal Sciences, for analysis. Approximately 15 g liver samples were frozen within 15 min after collection and stored at −18 °C for inductively coupled plasma mass spectrometry (ICP-MS) full scan element analysis and ^210^Po and ^210^Pb analysis.

**Table 1:** Ovine liver samples (*n* = 31) from South Greenland included in the case–control study

Sample ID^1^	Herd ID	Date of slaughter, 2021	Age group	Post-mortem finding	Liver cell pigment^2^	Kupffer cell pigment^3^
CA1	H2	09–23	Lamb	Acquired melanosis	2—3	0
CA2	H2	09–23	Lamb	Acquired melanosis	3	0
CA3	H7	09–27	Lamb	Acquired melanosis	2	0
CA4	H8	09–27	Lamb	Acquired melanosis	2	0
CA5	H5	09–27	Lamb	Acquired melanosis	4	0
CA6	H28	09–28	Lamb	Acquired melanosis	2—3	0
CA7	H28	09–28	Lamb	Acquired melanosis	2—3	0
CA8	H32	09–30	Lamb	Acquired melanosis	3	0
CA9	H35	09–30	Lamb	Acquired melanosis	2	+
CA10	H32	09–30	Lamb	Acquired melanosis	2	0
CA11	H32	09–30	Lamb	Acquired melanosis	5	+
CA12	H13	10–04	Lamb	Acquired melanosis	1	0
CA13	H10	10–05	Lamb	Acquired melanosis	2	0
CA14	H3	10–05	Lamb	Acquired melanosis	2	0
CA15	H3	10–05	Lamb	Acquired melanosis	3	0
CA16	H2	10–06	Sheep	Acquired melanosis	3	+
CA17	H9	10–06	Sheep	Acquired melanosis	3	+
CA18	H9	10–06	Sheep	Acquired melanosis	3	+
CA19	H4	10–06	Sheep	Acquired melanosis	2—3	0
CA20	H3	13–10	Sheep	Acquired melanosis	5	+
CA21	H36	10–25	Sheep	Acquired melanosis	3	+
CO1	H28	09–28	Lamb	No macroscopic changes	0	0
CO2	H28	09–28	Lamb	No macroscopic changes	1	0
CO3	H40	09–29	Lamb	No macroscopic changes	0	0
CO4	H40	09–29	Lamb	No macroscopic changes	0	0
CO5	H21	10–01	Lamb	No macroscopic changes	0	0
CO6	H8	10–06	Lamb	No macroscopic changes	0	0
CO7	H10	10–08	Lamb	No macroscopic changes	0	0
CO8	H12	10–08	Lamb	No macroscopic changes	0	0
CO9	H21	10–11	Lamb	No macroscopic changes	0	0
CO10	H1	10–13	Sheep	No macroscopic changes	1	+

^1^Cases: Sample ID CA1-21; Controls: Sample ID CO1-10

^2^Black pigmentation of hematoxylin and eosin-stained liver cells (hepatocytes) was graded visually: 0, not present; 1, very light; 2, light; 3, moderate; 4, marked; 5, severe

^3^Pigmentation of liver macrophages (Kupffer cells) was graded histologically (haematoxylin and eosin staining) as: 0, not present; +, present

Blood was collected from the heart ventricles and atria into 50 ml Nunc tubes during the post-mortem inspection using a pipette. The tubes were left overnight in a vertical position at 5 °C for clotting, followed by manual serum extraction and storage at −18 °C for subsequent analysis.

#### Histology

All formalin-fixed case and control liver samples were prepared for histopathology and stained with hematoxylin and eosin (HE) using standard methods (Bancroft & Layton, 2019).

Black pigmentation of the liver cells (hepatocytes) was graded visually in HE-stained sections using the following scale: 0, not present; 1, very light; 2, light; 3, moderate; 4, marked; 5, severe (Fig. 1d). Pigmentation of liver macrophages (Kupffer cells) was graded as 0, not present; +, present.

#### Serum biochemistry

The analyses of serum samples were conducted using an automated spectrophotometric analyser, which also contained Integrated Multisensor Technology for electrolyte analysis (Atellica Solution; Siemens Healthineers, Germany) at the Veterinary Diagnostic Laboratory at the Department of Veterinary Clinical Sciences, University of Copenhagen, Denmark. All serum samples (*n* = 31) were analysed within 2 months of sampling. Daily internal and quarterly external quality controls were performed on the selected assays. The biochemistry panel included the hepatic-related alanine aminotransferase (ALT), aspartate aminotransferase (AST), alkaline phosphatase, and gamma-glutamyl transferase (GGT). The biochemistry panel also included the hepatic/erythrocyte metabolic product total bilirubin, cholesterol as a lipid marker, the muscle enzyme creatinine kinase, and renal function parameters such as creatinine and urea. General protein parameters such as total protein, albumin, and globulins were analysed, along with the erythrocyte-related/acute-phase reactant iron.

Electrolytes such as inorganic phosphate, calcium, magnesium, sodium, and potassium were measured.

#### Element analysis

Each liver sample (cases, *n* = 21; controls, *n* = 10), with its unique ID and slaughter date listed in Table 1, was individually sealed in laboratory-grade plastic bags and stored at −18 °C from the time of collection until analysis. All case–control liver samples collected from South Greenland (Fig. 2) were analysed for element composition of 61 different elements at the accredited trace element laboratory at the Department of Ecoscience, Aarhus University, Roskilde, Denmark. A detailed description of the analytical method has been given in Søndergaard and Mosbech (2022). Briefly, sub-samples of approximately 1 g wet weight (w.w.) were cut from the main samples and microwave digested in an Anton Paar Multiwave 7000 oven in 4 mL/4 mL Merck Suprapure nitric acid/Milli-Q water. Digestion solutions were then diluted with Milli-Q water to c. 60 g and analysed using inductively coupled plasma mass spectrometry (ICP-MS), Agilent 7900 instrument (see Supplementary Material for raw ICP-MS data). As validation and to ensure analytical quality, blanks, sample duplicates, and the certified reference materials (CRM) DORM-4 (fish protein, *n* = 2) and DOLT-5 (fish liver, *n* = 1) from the National Research Council of Canada were analysed alongside the samples. The blanks were prepared using the same analytical procedure as for the sample preparation, but without the inclusion of healthy or black liver tissue. The detection limit (LD) of each element was calculated as 3 standard deviations on the series of blanks, and concentrations of elements below the LD were reported as < LD. The CRM data is reported in the “Supplementary information” section. The mean (± standard deviation) recovery of the elements in the CRMs (i.e. the accuracy) was 95 ± 5% for DORM-4 and 98 ± 15% for DOLT-5 for all certified element values and within 88–105% for the key elements As, Cd, Cu, and Zn (no certified values were available for Th and U). The laboratory is accredited by the Danish Accreditation Fund DANAK (no. 411) to analyses of Cr, Ni, Cu, Zn, As, Se, Cd, Hg, Pb, and U in biota with 15–35% precision (2 SD). All concentrations are reported in mg g^−1^ w.w., along with the DL for each chemical element (Supplementary Material for raw ICP-MS data).

#### Polonium-210 and lead-210 analysis

Polonium (^210^Po) was measured in fresh liver sample aliquots (*n* = 13 cases; *n* = 6 controls) by alpha spectrometry, using a modified version of the method by Chen et al. (2001) as described by Hansen et al. (2022). Activity concentrations are reported as Bq kg⁻^1^ wet weight (w.w.) with the associated standard deviation (SD) and were corrected for decay back to the sampling date. The SD reflects the experimental variability observed within each set of analysed samples. Analytical accuracy was ensured using an alpha spectrometry calibration standard source (^241^Am, ^244^Cm, and ^23^⁷Np), along with blank samples and a ^2^⁰⁹Po tracer yield determination. For ^210^Po, the minimum detectable activity (MDA) was 0.09 Bq kg⁻^1^, with a ^2^⁰⁹Po recovery of 75%, and a counting uncertainty (2σ) of 2.3 Bq kg⁻^1^. Lead-210 (^210^Pb) was analysed in some samples by following the analytical procedure described by Hansen et al. (2022).

#### Dose assessment

The annual absorbed dose to case and control livers, respectively, due to ^210^Po exposure, was calculated following the methodology described in Hansen et al. (2022). The annual average effective dose to adults, children, and most exposed persons in Greenland due to ingestion of ^210^Po in black and healthy livers, respectively, was estimated following the methodology described in Hansen et al. (2022). The range (minimum, maximum) and mean ^210^Po activity concentrations measured in both case and control liver samples were used to evaluate the influence of natural variability on the estimated annual average effective dose.

The annual consumption rates (kg y⁻1) of sheep liver with and without acquired melanosis for the average adult, child, and the most exposed individual were estimated based on liver consumption data from the 2018 Greenland Population Health Survey, with results weighted according to age, sex, and region of the Greenlandic population (Larsen et al., 2019). The annual average effective dose calculation was based on both the adult mean consumption and the mean consumption of the upper 10th percentile. The most exposed person is defined as belonging to this upper 10% consumption group (Hansen et al., 2022). The upper 10% group is defined as those participants who consumed more than the 90% percentile of sheep liver with and without acquired melanosis, respectively. The children’s consumption rate was estimated to be 50% of their parents’ (Nordic Council of Ministers, 2014; Hansen et al., 2022).

### Prevalence study

In December 2021, after termination of the slaughter season, the remaining sheep population in South Greenland consisted of 18,184 animals of which 14,778 were ewes. The present cross-sectional prevalence study included 20,115 animals from 44 different herds, 18,712 lambs, and 1403 sheep. These animals represented the entire Greenlandic sheep production slaughtered at the abattoir in Narsaq town from September 9 until October 27, 2021, except for sheep reared at a single farm near Nuuk. Based on an assumed acquired melanosis prevalence of 0.25 judged from previous observations at the abattoir in Narsaq, the targeted sample size was 462 with a precision of 0.05, a confidence interval of 0.95, and test sensitivity and specificity of 0.9 (Sergeant, 2018, Epitools, analysed February 13, 2025). Consequently, the study included a significantly larger number of animals than the targeted sample size, ensuring highly accurate and reliable prevalence estimates. All animals were identified by an individual ear tag (animal id), herd id (and thus grazing area), and slaughter date. Age (lamb or sheep), slaughter weight, carcass quality, i.e. fat- and meat/muscle content, and diseases, including acquired melanosis found at the post-mortem inspection, were registered by the abattoir for all animals.

The overall prevalence of acquired melanosis in Greenlandic sheep (*n* = 1403) and lambs (*n* = 18 712) was determined by dividing the number of livers with acquired melanosis detected during post-mortem inspections by the total number of slaughtered animals, including both age groups. Additionally, the prevalence was calculated separately for lambs and sheep by dividing the number of acquired melanosis cases in each age group by the total number of slaughtered animals in that age group. Similarly, the herd prevalence for each farm was calculated by dividing the number of acquired melanosis cases at the farm level by the number of slaughtered animals from each farm. The confidence intervals were calculated according to Rogan and Gladen (1978).

#### Acquired melanosis, grazing area, and herd

Data registered by the abattoir were used for descriptive statistics, thus presenting histograms of acquired melanosis frequencies according to grazing area and herd for both lamb and sheep.

#### Correlation between acquired melanosis, carcass quality (fat- and muscle/meat content), and other markers of disease

As part of the slaughter process, the carcasses were trimmed for abdominal fat before weighing, and the carcass quality score was registered by a trained employee. Briefly, a visual inspection of the carcass determines a score/rank of the fat- and the muscle/meat content. This score is a two-digit number, of which the first digit is the muscle/meat content scored relatively from 1 (high) to 5 (low), and the second digit is the fat content scored relatively from 1 (low) to 5 (high). A high muscle/meat and a moderate fat content is preferable, i.e. a score of 12 will give the highest revenue to the farmer. The scores were used as a health indicator, and the possible correlations between the scores and acquired melanosis, chronic infections (i.e. chronic pneumonia, pleuritis, peritonitis, and arthritis), and cachexia (emaciation, morbid loss of weight) were analysed as described in section “Statistical analysis”.

### Statistical analysis

Statistical differences in element concentrations (i.e. As, Cd, Cu, Zn, Th, U, ^210^Po, ^210^Pb) between acquired melanosis (black livers) and the control group (normal livers) were estimated using two-sample *t*-tests. Before conducting the *t*-tests, the Shapiro-Wilks test was applied to assess the normality of the data, and Levene’s *F* test was employed to evaluate the homogeneity of variance amongst groups. If assumptions were not satisfied, differences between acquired melanosis and control groups were assessed using the Wilcoxon rank-sum exact tests. The same statistical procedure was used to test for differences in element concentrations between lamb and sheep livers but only considering those animals with acquired melanosis (black livers). Before statistical analysis, concentrations of both non-radioactive and radioactive elements were log-transformed to approach normality and homoscedasticity. Analysis of variance (ANOVA) was also performed to test the difference in element composition (Se, F, Ag, Pb, Hg) between acquired melanosis (black livers) and the control group (normal livers) and grazing areas. Multivariate analyses (PCA) were performed to test possible correlations between Cu concentrations and Se, Fe, Ag, Pb, and Hg. ANOVA and PCA were performed using the group variable case vs. control and only chemical elements of which concentration was 80% above the detection limit, see ICP-MS raw data, Supplementary Material.

Differences in serum biochemistry parameters were estimated between lamb and sheep as well as between animals with acquired melanosis (case) and control animals. The latter test was repeated following the exclusion of three animals with an uncertain classification of disease status, i.e. very light pigmentation detected by histology (CA12, CO2, CO10). Differences between age classes and case vs. control animals were quantified using either Student’s *t*-test or Welch *t*-statistic, depending on whether the groups had equal variances as determined by Fisher’s *F*-test.

Multi-model inference of black liver prevalence as a function of all elements and ^210^Po and ^210^Pb concentrations was performed. The final average model was adjusted for multicollinearity (i.e., only included models with rs < 0.5). Steps 1–2 were repeated for each age class.

Age (sheep versus lambs) as a risk factor for the presence of pigmented Kupffer cells in the livers was tested using a chi-square test (Yates correction for small numbers).

To assess whether acquired melanosis, chronic infection, and cachexia can affect the quality in terms of fat and meat of lamb and sheep carcasses, the data were analysed using generalised linear mixed models (GLMM). The fat rank and meat rank indices were fitted as the response variables in separate GLMMs, while acquired melanosis, chronic infection, and cachexia were fitted as categorical predictor variables (labelled as yes or no) in all GLMMs. As such, no variable selection was required for the GLMMs as all variables were needed to address the research question. To account for potential variation in meat or fat classification unrelated to acquired melanosis, chronic infection, or cachexia, the slaughter week was modelled as a random effect nested within herd ID and grazing area, reflecting the origin of each lamb or sheep. Including slaughter weight as a random effect also incorporates any potential confounding effect of time.

## Results and discussion

### Case–control study

#### Diagnosis of acquired melanosis and serum biochemistry

An example of a liver with acquired melanosis is shown in Fig. 1b. The colour of livers with acquired melanosis varied from greyish to dark black, and a distinct reticular pattern was frequently observed (Fig. 1c). Dark pigments were visible in the periportal and midzonal liver cells (hepatocytes) (Fig. 1d), corresponding to the reticular pattern seen macroscopically. Table 1 shows the results of the histological pigment grading in the 31 samples. Pigmentation was evident in the Kupffer cells of several livers, and this pigment had the same tinctorial properties as that found in the hepatocytes. When these pigmented, phagocytic, often multinuclear cells were present, they appeared to be more abundant towards the central vein, i.e. in the direction of the blood flow, thus indicating a clearance mechanism simultaneously with the formation of the pigments. Furthermore, a chi-square test for age (sheep versus lambs) as a risk factor for the presence of pigmented Kupffer cells was 6.6 (*p* < 0.01). Thus, pigmented Kupffer cells were more frequent in sheep than in lambs, indicating a time-dependent process of pigment accumulation and clearance. In two out of 10 macroscopically normal livers (controls), we observed a very light accumulation of pigments in the liver cells, while in one of the affected livers (cases), only a very light accumulation of pigments was observed histologically. These observations correspond to the variation seen macroscopically (greyish to dark black) and further indicate a time effect on accumulation/clearance. It is important to note that small sample sizes limit the statistical power of our conclusions regarding age-related pigment accumulation in hepatocytes and Kupffer cells.

Serum biochemistry analysis revealed significantly elevated cholesterol levels in cases compared to the controls (*p* = 0.046). Excluding the three samples with uncertain classification (CA12, CO2, CO10) accentuated this difference (*p* = 0.005). Additionally, sheep, irrespective of melanosis status, exhibited higher cholesterol levels than lambs (*p* = 0.0003) (Table 2S, Serum Biochemistry Results, Supplementary Material). This observation may indicate a potential impact on liver function due to acquired melanosis (Alemu et al., 1977). However, other key liver enzymes, including ALT, AST, and GGT, remained unaffected by the presence of acquired melanosis.

Few studies have included blood markers of liver lesions. Cullen and Stalker (2015) do not link acquired melanosis with liver lesions. Given the limited number of animals, particularly adult sheep, included in our case–control study, these findings should be interpreted with caution. Further research is necessary to determine whether acquired melanosis influences liver function.

The macroscopic appearance and histology of the livers with dark discoloration corresponded to acquired melanosis or hepatic lipofuscinosis, as described by Winter (1961) and Nordstoga (1990), respectively.

#### Element concentrations

Kvanefjeld deposit in southern Greenland, located 7 km from Narsaq town, includes REE as lanthanum, cerium, neodymium, praseodymium, europium, dysprosium, terbium, and yttrium, as well as uranium, thorium, zinc, and fluor. Analytical results for all 61 analysed elements of case–control liver samples are shown in the ICP-MS raw data, Supplementary Material. In both cases and controls, REE concentrations are near the detection limit, and some values fall below the detection limit (ICP-MS raw data, Supplementary Material). Therefore, REEs are not included in the following discussion. The discussion below focuses on selected toxic and essential elements, specifically arsenic (As), cadmium (Cd), copper (Cu), zinc (Zn), uranium (U), and thorium (Th), due to their environmental relevance, potential biological effects, and their association with the Kvanefjeld deposit located near Narsaq town and the slaughterhouse. Arsenic and cadmium are toxic heavy metals, and uranium and thorium are elements associated with the Kvanefjeld exploration project, all posing toxicological risks to both humans and animals. Copper, while essential, can be toxic to sheep due to their limited hepatic storage capacity (Gupta, 2018). Zinc is a critical micronutrient involved in enzymatic processes and is indispensable for healthy growth and reproduction in both plants and animals (Suttle, 2010). These elements were therefore selected for detailed examination in relation to potential exposure pathways and health implications.

**Fig. 3:**
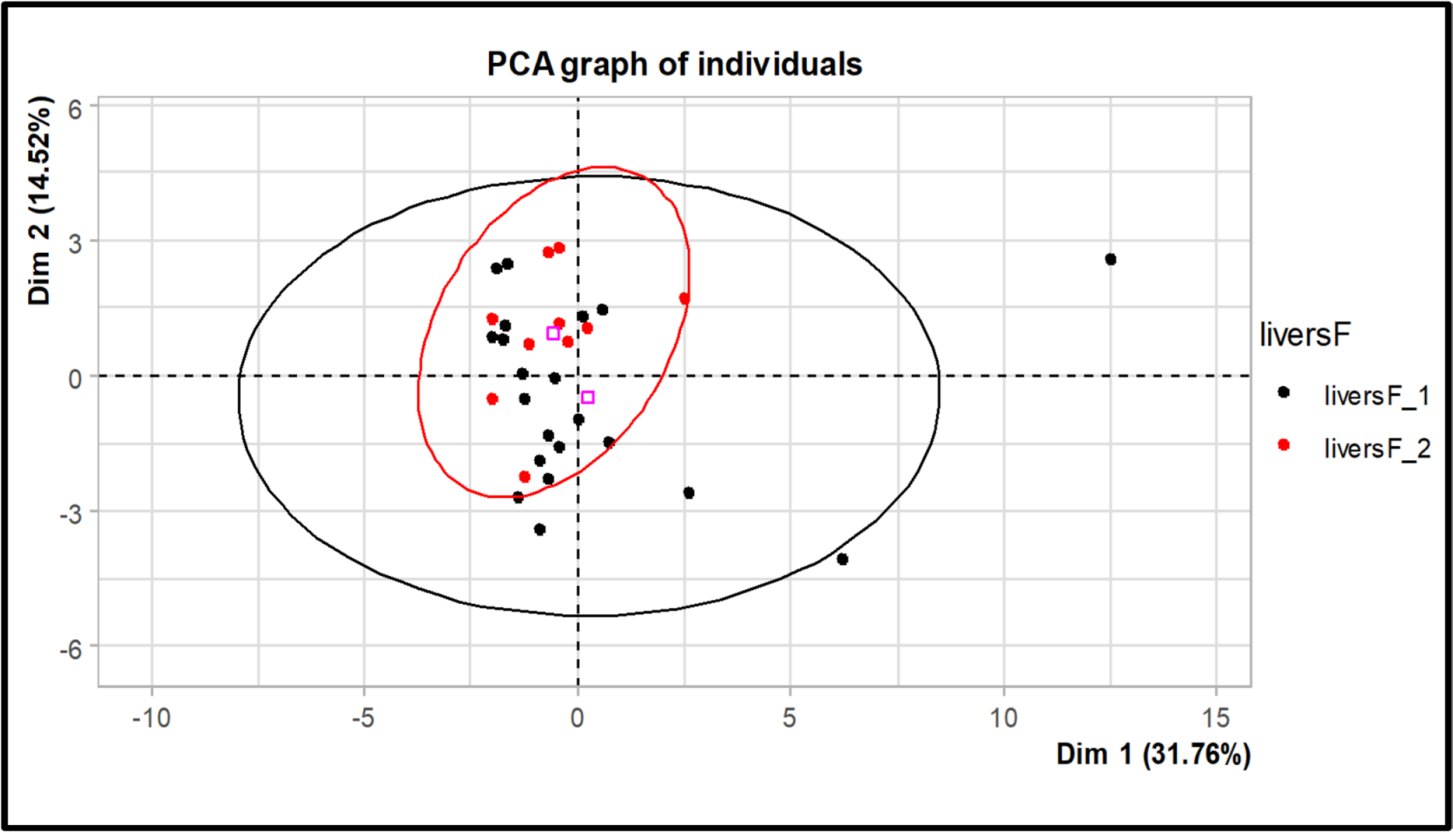
Principal component analysis (PCA) graph of individuals showing no statistical difference in chemical elements between acquired melanosis (black) and the control group (red) of analysed sheep and lambs

No significant statistical differences (*p*-value = 0.639) in the selected 29 element concentrations between case and control livers in analysed sheep and lambs were detected (Fig. 3, ICP–MS raw data, Supplementary Material). Further, no statistical differences in element concentrations between case and control livers were observed between grazing areas (*p*-value = 0.633).

Although the statistical tests were appropriately applied, the sample sizes for both groups (acquired melanosis, *n* = 21; controls, *n* = 10) limit the analysis’s statistical power. Consequently, our results should be interpreted with caution, as subtle differences may remain undetected.

#### Arsenic

The inorganic form of arsenic is known to be highly toxic (World Health Organization, 2022). Arsenic can be present in the diet through meat, poultry, dairy products, and cereals; however, the levels of exposure from these foods are generally much lower compared to those from contaminated groundwater. No statistical differences (Wilcoxen test, *p*-value = 0.835) in arsenic concentrations were detected in the case (acquired melanosis - black livers) and control (normal livers) groups, with concentrations ranging from 0.004 to 0.007 mg kg^−1^ w.w., with most samples containing arsenic levels below the LOD of 0.003 mg kg^−1^ w.w. (ICP-MS raw data, Supplementary Material). In acquired melanosis livers (black livers), concentrations of As were statistically higher (Wilcoxon test, *p*-value = 0.002) in sheep than lambs. Arsenic concentrations measured in the livers, both in cases and controls, were below the average concentrations reported for Australian sheep livers of 0.01 mg kg⁻^1^ w.w. (MacLachlan et al., 2016).

#### Cadmium

Cadmium is a naturally occurring toxic heavy metal, with dietary intake representing the primary exposure pathway for non-smokers (European Food Safety Authority, 2012). Over 90% of the cadmium present in the surface environment originates from anthropogenic activities, particularly agricultural and industrial operations. Potential anthropogenic sources of cadmium exposure for farmed ruminants include coal and oil combustion, mining, metal smelting, alloy production, and other industrial processes involving cadmium. Exposure concentrations typically decline with increasing distance from the contamination source (Pan et al., 2010; Swarup et al., 2007; Vos et al., 1988).

Our results show no statistical differences (*t*-test, *p*-value = 0.351) in mean Cd concentrations between case and control livers. In case livers (acquired melanosis - black livers), concentrations of Cd were statistically higher (Wilcoxon test, *p*-value = 0.005) in sheep than in lambs. In lambs, Cd concentrations in case livers were 0.13 mg kg^−1^ w.w. (*n* = 15), and in controls, 0.13 mg kg^−1^ w.w. (*n* = 9). In case livers from sheep (*n* = 7) and controls (*n* = 1), the concentrations ranged from 0.1 to 1.6 mg kg^−1^ w.w. with a mean of 0.5 mg kg^−1^ w.w. MacLachlan et al. (2016) documented cadmium levels in tissues of Australian sheep, with concentrations varying from below 0.003 up to 11.7 mg kg⁻^1^ w.w. The highest cadmium concentration was found in the kidney (average 0.85 mg kg⁻^1^ w.w.), followed by the liver (average 0.3 mg kg⁻^1^ w.w.) and muscle tissue (average 0.006 mg kg⁻^1^).

Cadmium limits are established in various countries mainly to safeguard human health rather than animal health. A critical cadmium intake dose of 2.5 mg per kg of body weight per day in sheep, sustained over 1 year, was found to cause chronic toxicity and subclinical effects (Wilkinson et al., 2003). According to a report by the European Food Safety Authority (2004), clinical symptoms in sheep are unlikely to occur with a daily cadmium intake below 5 mg per kg of feed. However, adverse effects have been reported in ruminants at cadmium levels lower than this threshold. Heffron et al. (1980) observed liver cell degeneration in sheep fed corn silage with a cadmium concentration of 1.7 mg kg⁻^1^ dry matter. However, the minimum toxic dose and the maximum safe dietary levels of cadmium for ruminants remain unclear. To safeguard human health, the European Union has set maximum allowable cadmium concentrations in complete and complementary feeds for ruminants at 1.0 mg/kg and 0.5 mg/kg, respectively, with a moisture content of 12% (European Commission Directive 2002/32/EC). Additionally, the maximum cadmium concentration limits permitted in cattle muscle, liver, and kidney tissues intended for human consumption are regulated by European Commission Regulation 1881/2006 (as amended by Regulation No. 629/2008), with limits of 0.05, 0.5, and 1.0 mg/kg w.w., respectively. Phillips and Tudoreanu (2011) developed a model estimating cadmium concentrations in sheep liver and kidneys by considering soil and diet factors. Their model predicts that, with soil cadmium levels at 1 mg kg⁻^1^ dry weight, cadmium concentration in the kidneys could exceed 1 mg kg⁻^1^ after 150 days of grazing supplemented with additional feed.

The Joint FAO/WHO Expert Committee on Food Additives (n.d.) set a provisional tolerable monthly intake of 0.025 mg kg^−1^ body weight, while the European Food Safety Authority Panel on Contaminants in the Food Chain recommended a tolerable weekly intake of 0.0025 mg kg^−1^ body weight (European Food Safety Authority, 2012). In Greenland, the annual consumption of liver is 2 kg and 2.3 kg, respectively, for the average adult and the most exposed person (10% percentile). For children, the annual average consumption rate of liver is set to half that of their parents. Considering the annual average liver consumption rates for adults, children, and the most exposed person, along with the cadmium concentrations in sheep livers with and without acquired melanosis, it can be concluded that the recommended provisional tolerable monthly intake is not exceeded.

#### Copper

No statistically significant differences in Cu concentrations were observed between case and control liver samples (*t*-test, *p* = 0.653), nor between livers with acquired melanosis (black livers) in sheep and lambs (*t*-test, *p* = 0.277). Analysis of variance shows no significant difference in Cu between sub grazing areas (*p* = 0.433). Concentrations of Cu in lamb livers ranged from 36.0 mg kg⁻1 wet weight (w.w.) in case livers (*n* = 15) to 48.0 mg kg⁻1 w.w. in control livers (*n* = 9). In sheep, Cu concentrations varied from 66.0 mg kg⁻1 w.w. in case samples (*n* = 7) to 75.0 mg kg⁻1 w.w. in control (*n* = 1) (Supplementary Material for raw data).

Our results are comparable with published Cu concentrations in livers from healthy lambs and sheep. MacLachlan et al. (2016) reported Cu concentrations in Australian sheep liver at 66.0 mg kg^−1^ w.w. A mean copper concentration of 65.2 mg kg⁻^1^ wet weight was found in sheep livers collected between 1975 and 1983 from all Australian states (Langlands et al., 1987). Similarly, Kolbaum et al. (2023) reported Cu concentrations in German sheep liver of 73 mg kg^−1^ w.w. Sivertsen and Plassen (2004) measured a mean copper concentration of 69 mg kg⁻^1^ w.w. in lamb livers collected from Norway. The copper concentration in the liver can serve as an indicator of both copper deficiency and toxicity in sheep populations (Hill & Shannon, 2019). Hosking et al. (1986) describe chronic Cu toxicity in sheep associated with ingesting plant-based hepatotoxins or grazing pastures with high clover content. Liver damage caused by pyrrolizidine alkaloids, such as those found in Heliotropium species, is correlated with increased Cu accumulation within the liver. Copper over-supplementation through forage or concentrate can lead to chronic toxicity. Lambs and sheep in this study did not exhibit liver copper concentrations exceeding 337 mg kg^−1^, which is indicative of toxicity (MacLachlan et al., 2016).

**Fig. 4:**
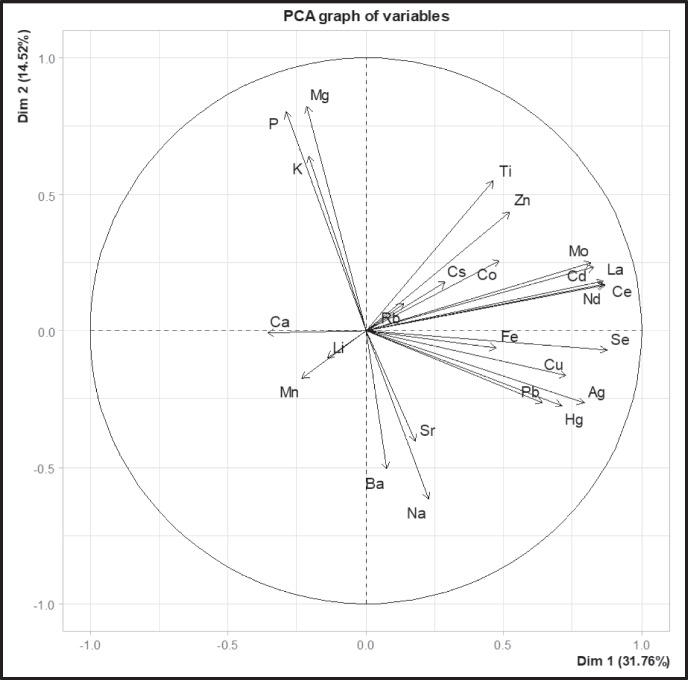
Cu positive correlation with elements such as Se, Fe, Ag, Pb, and Hg

Our results show that Cu correlate positively with Se (*F* = 0.1501, *P* < 0.7013), Fe (*F* = 0.891, *P* < 0.353), Ag (*F* = 1.696, *P* < 0.2031), Pb (*F* = 6e-04, *P* < 0.9814), and Hg (*F* = 0.1941, *P* < 0.6628) in Greenlandic lamb and sheep (Fig. 4). The average Se concentration in case livers of lambs was 0.08 mg kg^−1^ (w.w., *n* = 15), while control livers of lambs had a concentration of 0.1 mg kg^−1^ (w.w., *n* = 9). In sheep, the Se concentration was 0.3 mg kg^−1^ (w.w.) in case and control livers, which is comparable to 0.31 mg kg^−1^ (w.w.) reported in Australian sheep livers (MacLachlan et al., 2016). In lambs, the Fe average concentration was 64.2 mg kg^−1^ (w.w.) in case livers and 80 mg kg^−1^ (w.w.) in the controls. In sheep, the Fe concentration ranged from 47.2 mg kg^−1^ (w.w.) in cases to 141.4 mg kg^−1^ (w.w.) in the control. Average Ag concentrations were 0.2–0.3 mg kg^−1^ (w.w.) in lambs and 0.6–1.1 mg kg^−1^ (w.w.) in sheep in cases and controls.

The average concentrations of Pb varied between 0.01 and 0.02 mg kg^−1^ (w.w.) and Hg varied between 0.01 and 0.03 mg kg^−1^ (w.w.) in case and control samples from sheep and lambs. Reported mean Pb and Hg concentrations in Australian sheep livers were 0.04 and 0.003 mg kg^−1^ (w.w.), respectively (MacLachlan et al., 2016).

#### Zinc

Our study found no statistically significant differences in zinc (Zn) concentrations between case and control livers (*t*-test, *p* = 0.170), nor between livers exhibiting acquired melanosis (black livers) in sheep and lambs (*t*-test, *p* = 0.277). The mean concentrations of Zn in the present study varied between 30 and 33 mg kg^−1^ (w.w.) in lamb and 34 and 39 mg kg^−1^ (w.w.) in sheep livers, both in cases and controls. Zinc concentrations measured in this study fall within the range reported in previous studies. MacLachlan et al. (2016) found Zn concentrations of 37.2 mg kg^−1^ (w.w.) in livers from Australian sheep.

#### Uranium and thorium

The Kvanefjeld deposit near Narsaq town contains REE, U, Th, Zn, F, and other minerals. Exploration activities and the possibility of future mining have raised public concern that such activities could adversely impact local sheep farming and pose environmental risks due to potential exposure to U, Th, and associated elements.

No statistically significant difference in U concentrations was observed between case and control liver samples (*t*-test, *p* = 0.286) (ICP–MS raw data, Supplementary Material). In the present study, U concentrations in case and control livers from lambs varied from 0.00003 to 0.001 mg kg^−1^ (w.w.) with most samples containing uranium at concentrations below the LOD of 0.00003 mg kg^−1^ (w.w.). In case and control livers from sheep, the uranium concentrations were 0.0004 mg kg^−1^ (w.w.) and 0.00006 mg kg^−1^ (w.w.), respectively. Concentrations of U were significantly higher in case livers from sheep (*n* = 7) compared to those from lambs (*n* = 16) (Wilcoxon test, *p* = 0.004). These findings should be regarded as preliminary, consistent with the pilot nature of the study and the limited number of case samples. Samuel–Nakamura et al. (2017) reported uranium concentrations of 0.001 mg kg⁻1 dry weight in sheep livers collected from a mining-affected region in the northwestern part of the Navajo reservation, New Mexico. Limits for radionuclide concentrations in foods meant for human consumption and international trade are established in the Codex Alimentarius (Codex, 2013). Concentration limits in the Codex Alimentarius (2013) are established based on an annual intervention exemption level of 1 mSv. For uranium-235, a concentration of 100 Bq kg⁻1 is specified, excluding uranium-238 (Codex, 2013).

No statistically significant differences in concentrations of Th were observed between case and control liver samples (*t*-test, *p* = 0.346), nor between livers with acquired melanosis (black livers) in sheep and lambs (*t*-test, *p* = 0.286). Thorium concentrations in both case and control livers of lambs in the present study were below the LOD of 0.0001 mg kg^−1^ (w.w.). In sheep, all samples were also below the LOD, except for two samples (ranging from 0.0001 to 0.0004 mg kg^−1^ w.w.).

#### Activity concentration of ^210^Po and ^210^Pb

No statistical differences (Wilcoxon test, *p*-value = 0.291) in polonium-210 activity concentrations were found between the case and control livers of lambs and sheep. The relatively small sample sizes for the acquired melanosis group (*n* = 13) and the control group (*n* = 6) constrain the statistical power of the analysis, and the findings should therefore be interpreted with caution. Activity concentrations of ^210^Po and ^210^Pb in case and control livers of lambs and sheep are summarised in Table 2. Sheep case livers had statistically higher ^210^Po activity concentrations compared to lamb case livers (*t*-test, *p*-value = 0.02). Mean ^210^Po activity concentrations in case livers from lambs (*n* = 7) were 12.3 ± 5.5 Bq kg^−1^ w.w., ranging from 2.5 to 21.0 Bq kg^−1^ w.w. In the corresponding control livers (*n* = 5), the mean ^210^Po activity concentrations were 22.2 ± 13.0 Bq kg^−1^ w.w., varying from 16.0 to 48.4 Bq kg^−1^ w.w. In the case livers from sheep (*n* = 6), the mean ^210^Po activity concentrations were 36.0 ± 12.2 Bq kg^−1^ w.w., ranging from 13.0 to 49.0 Bq kg^−1^ w.w. Activity concentration of ^210^Po in the sheep control liver (*n* = 1) was 19.4 Bq kg^−1^ w.w. (Table 2).

**Table 2:** Activity concentrations of ^210^Po and ^210^Pb (Bq kg^−1^ w.w.) and derived annual absorbed dose (µGy/year) from exposure to ^210^Po

Common name	Liver sample	Number of samples	^210^Po mean ± *SD*	^210^Po min.—max	Counting uncertainty (2 *σ*) (Bq kg^−1^)	Number of samples	^210^Pb mean ± *SD*	^210^Pb min.—max	Counting uncertainty (2 *σ*) (Bq kg^−1^)	Absorbed dose mean ± *SD*	Absorbed dose min.—max
Lamb	Case: Acquired melanosis	7	12.3 ± 5.5	2.5–21.0	± 1.4	1	10 ±	± 10	± 1.4	336 ± 150	68–566
Sheep	Case: Acquired melanosis	6	36.0 ± 12.2	13.0–49.0	± 3.1	2	9.8 ± 2.0	8.4–11.1	± 1.45	982 ± 335	346–1326
Lamb	Control: Healthy livers	5	22.2 ± 13.0	16.0–48.4	± 2.7	2	8.3 ± 0.3	8.3–8.34	± 1.2	607 ± 354	436–1322
Sheep	Control: Healthy livers	1	19.4 ±	− 19.4	± 2.3	1	10.4 ± 0.7	− 10.4	± 1.2	530 ±	± 530

Activity concentrations of ^21^⁰Pb were lower than those of ^21^^0^Po, and no statistical differences between case and control livers of lambs and sheep were observed (Wilcoxon test, *p* = 0.629) (Table 2). The derived annual ^210^Po absorbed dose to case and control livers (Table 2) is several orders of magnitude lower than the default ERICA whole-body screening dose rate of 10 µGy/h (ERICA tool, n.d.).

#### Dose assessment

**Fig. 5:**
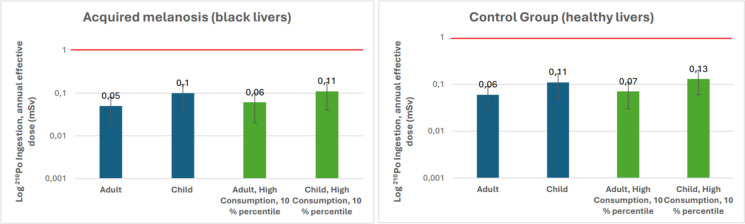
Annual average effective dose (µSv) due to ingestion of ^210^Po to children, adults, and the most exposed person (10% percentile). The red line indicates the annual reference dose of 1 mSv recommended by IAEA (2014)

Figure 5 presents the annual average effective dose from ingestion of ^210^Po in livers with and without acquired melanosis for children, adults, and most exposed individuals in Greenland. The mean annual average effective dose from consuming ^210^Po in livers without acquired melanosis was estimated at 60 ± 0.03 µSv for adults and 110 ± 0.06 µSv for 5-year-old children.

In this study, the individuals most exposed are those with the highest annual liver consumption rates who reside in settlements. They are defined as the 10th percentile of the Greenlandic population. The annual average effective doses from ^21^^0^Po in consumed livers without acquired melanosis to the most exposed adults and children range between 70 and 130 µSv (Fig. 5).

In Greenland, the annual average effective doses from ingestion of ^21^^0^Po in liver tissue, with or without acquired melanosis, to children, adults, and the most exposed individuals are lower than the estimated doses from ^21^^0^Po intake via seafood and marine mammals consumption, which are 1.4 mSv y⁻¹ for children, 2 mSv y⁻¹ for high-consuming adults, and 3.6 mSv y⁻¹ for high-consuming children (Hansen et al., 2022). The annual average effective dose from ingesting ^210^Po in healthy livers to the most exposed children (10% percentile) is slightly higher than the world annual average effective dose of 120 µSv (UNSCEAR, 2000) from ingesting naturally occurring radionuclides, excluding ^40^K.

The International Commission on Radiological Protection (ICRP, 2007) recommends an annual effective dose to the public not exceeding 1 mSv (1000 µSv) above natural background radiation for planned industrial activities. A reference dose of 1 mSv y^−1^ from food consumption is recommended for existing exposure situations (IAEA, 2014). Greenland’s public is exposed to less than 1 mSv y^−1^ through the ingestion of ^210^Po in sheep and lamb livers (Fig. 5).

Sheep and lamb livers analysed in this study were fresh. If the Greenlandic population cooks livers before eating, some ^21^^0^Po may be lost due to its volatilization at approximately 80 °C. Literature studies report varying effects of cooking on ^21^^0^Po concentrations, with some finding notable losses while others observe increases, depending on the food type and cooking technique (Komperød et al., 2020; Štrok & Smodiš, 2011; Uddin et al., 2019).

Due to mixed results reported in the literature, no adjustment was made to the ^210^Po concentrations used to estimate doses to children, adults, and the most exposed individuals in Greenland. If sheep and lamb livers are frozen and consumed several months after slaughter, such as 6 months later, ^21^^0^Po will decay due to its relatively short half-life of 138 days. There is currently no available data on the time between slaughter and liver consumption in Greenland. As a result, no adjustments were made when estimating the annual ^210^Po doses to adults, children, and individuals most exposed. This lack of information introduces uncertainties in the derived dose from ^210^Po ingestion and highlights the need for further research on cooking effects.

### Prevalence

#### Prevalence of acquired melanosis, grazing area, and herd

**Fig. 6:**
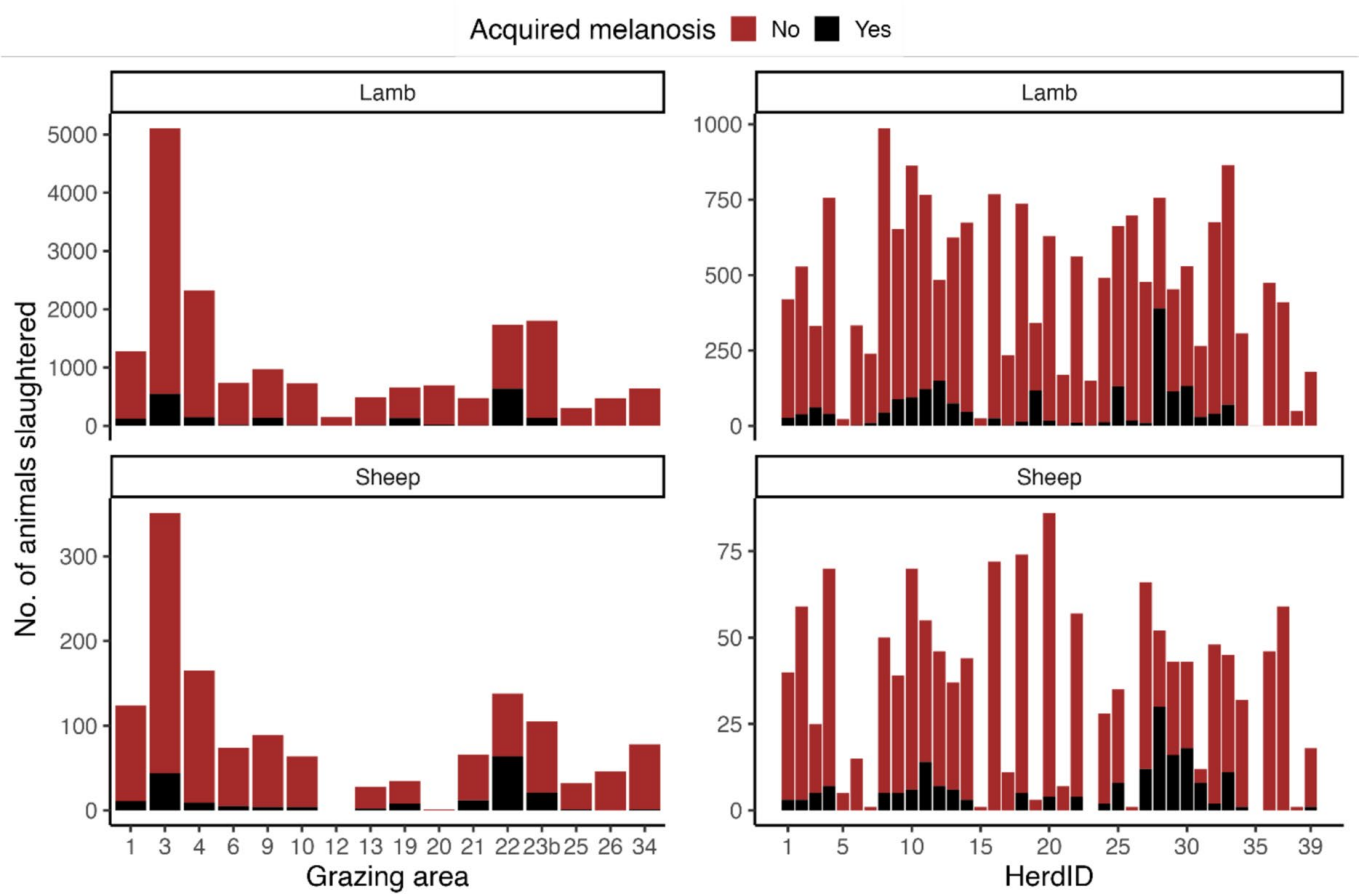
Variation in the numbers of animals with acquired melanosis across the different farms: left panel, number of acquired melanosis cases registered across age class (lamb or sheep) and grazing area, and right panel, across age (lamb or sheep) and herd (HerdID)

In 2021, the overall prevalence of ovine acquired melanosis in South Greenland was 10.6% (95% CI 10.1%–13.2%), and the age-specific prevalences were 10.4% (95% CI 9.9%–13.0%) and 13.4% (95% CI 12.1%–17.5%) for lambs and sheep, respectively. The number of animals with acquired melanosis according to age class and grazing area, and age class and herd ID, is shown in Fig. 6a, b, respectively. Prevalence across grazing areas and herds is shown in Fig. 2. The herd prevalence ranged from 0% (95% CI 0.0%–0.0%) to 51.8% (95% CI 48.6%–64.8%), with the highest prevalences clustering in grazing area 22.

In contrast, herds located in the southernmost parts of the region (grazing areas 25, 26, and 34) had prevalences below 1.5%. The reasons for this uneven distribution were not investigated in the present study.

Meat and Livestock Australia homepage (2018) reports that acquired melanosis is commonly observed as an incidental finding during slaughter in apparently healthy lambs and sheep. The condition is generally observed in livestock that graze on certain types of pastures in regions such as Australia, the Falkland Islands, and Norway. Prevalence studies are scarce, and most reports are based on the registration of abattoir findings. According to Winter (1961), a prevalence of around 3% was estimated in abattoirs in Brisbane, Australia, in the 1960s, and McCrea (1970) noticed, without presenting any data, “that dark pigmentation of the liver” was widespread in sheep on the Falkland Islands in the 1970s. Cullen and Stalker (2015) reported acquired melanosis as a condition of adult sheep only. To the best of our knowledge, this study is the first to systematically document all cases of acquired melanosis in a large population and over an entire slaughter season, enabling the determination of the true prevalence of ovine acquired melanosis in Greenland.

#### Correlation between acquired melanosis and carcass quality: fat and muscle/meat content and other markers of disease

**Fig. 7:**
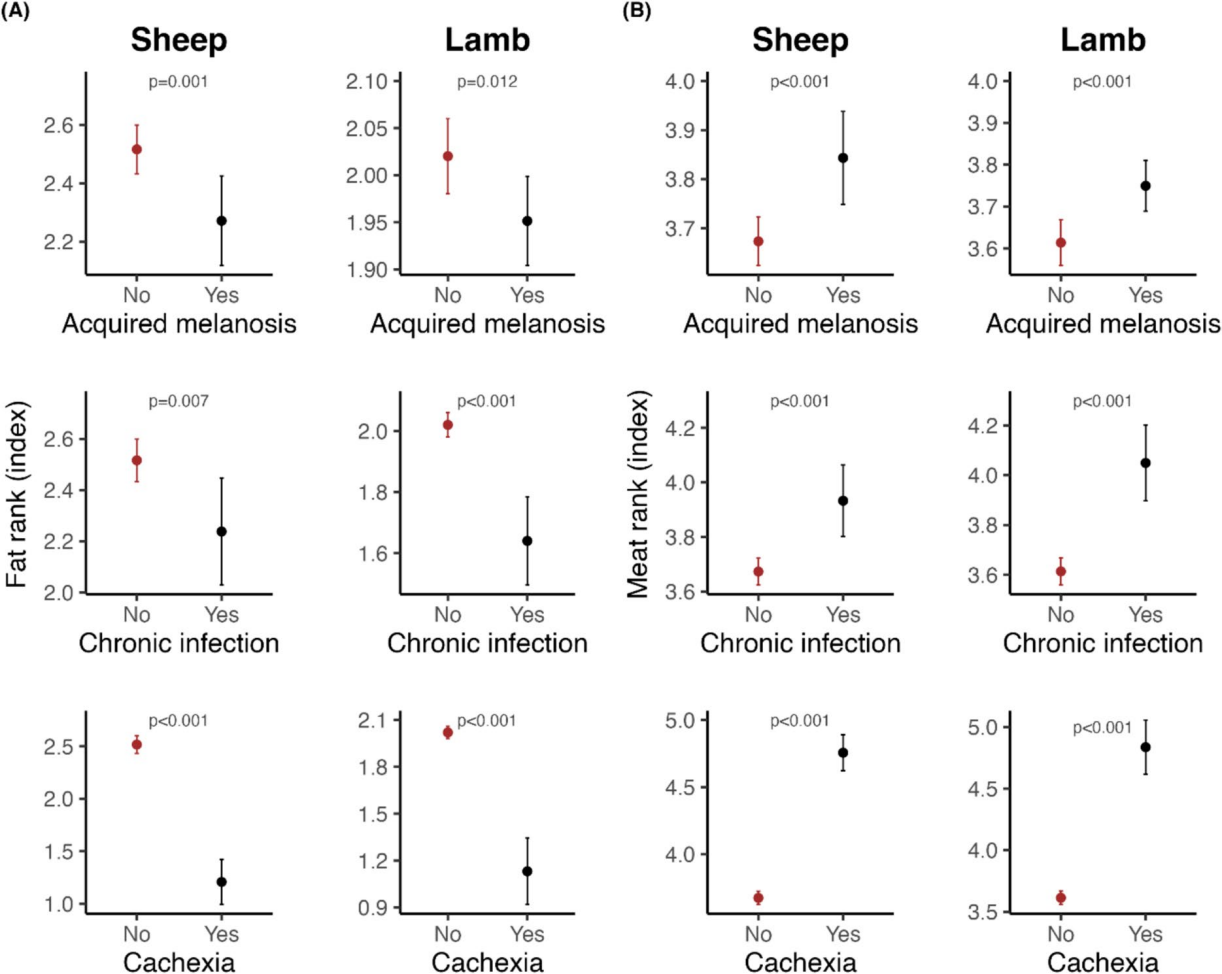
**A** Association between acquired melanosis (top panel), chronic infection (middle panel), and cachexia (bottom panel) and fat rank (range from 1 (low) to 5 (high) for sheep and lambs), and **B** association of acquired melanosis (top panel), chronic infection (middle panel), and cachexia (bottom panel) and muscle/meat rank (range from 5 (low!) to 1 (high!) for sheep and lambs

Figure 7 illustrates a significant and pronounced reduction in both fat- and muscle/meat content associated with chronic infection and cachexia, the latter defined as severe pathological weight loss. Chronic infections typically weaken the animals, reducing their ability to withstand harsh conditions in mountain pastures, which negatively affects fat and muscle/meat deposition at slaughter. A slight but statistically significant decrease in fat and muscle/meat content was also observed in animals with acquired melanosis, both lambs and sheep. However, this does not necessarily imply a causal relationship. Histological findings reported in section “Diagnosis of acquired melanosis and serum biochemistry” suggest a dynamic mechanism of pigment accumulation and clearance. Due to the very limited number of animals affected by chronic infection or cachexia, we were unable to directly test for associations between these conditions and acquired melanosis.

Potential registration errors at the abattoir could have influenced the results; however, we consider this risk negligible, as such random errors are likely to occur in both directions and thus have no systematic effect. Similarly, the quality of disease indicator data and carcass quality assessments depended on evaluations performed by veterinarians and a specially trained technician, respectively. To ensure consistency, several carcasses were re-examined on multiple occasions. Nevertheless, meat inspection conducted by veterinarians inherently carries a risk of human error, the magnitude of which could not be quantified in this study.

## Limitations and future perspectives

This case–control pilot study was initiated in response to public concerns about potential environmental impacts from exploration activities at the Kvanefjeld deposit in South Greenland. The study provides valuable new insights into acquired melanosis in sheep. However, the relatively small sample size (21 cases and 10 controls) and the uneven distribution between lambs and adult sheep limit the generalizability of the findings. Future research should include a larger and more representative sample, encompassing diverse herds across Greenland and ensuring a balanced age distribution. We recommend prioritising case–control studies that target herds with the highest and lowest observed prevalences.

In addition, future studies should incorporate farm management practices, grazing area characteristics, risk factor assessment, and pigment composition analysis. Such an approach could provide a more comprehensive understanding of the potential causes underlying hepatic acquired melanosis in sheep, thereby enabling a more robust biological and ecological interpretation.

## Conclusions

The macroscopic appearance and histology of the black livers demonstrated the presence of acquired melanosis (syn. hepatic lipofuscinosis). Serum biochemistry, specifically liver enzyme analysis, indicated elevated cholesterol levels in livers affected by acquired melanosis (cases). However, given the small sample size in the case–control study and the normal values for other liver enzymes, we cannot draw definitive conclusions regarding the potential impact of acquired melanosis on liver function.

For the first time, the true prevalence of ovine acquired melanosis was determined with high precision based on a large sample size. The overall prevalence was estimated at 10.6% (95% CI 10.1%–13.2%), with herd prevalences ranging from 0% (95% CI 0.0%–0.0%) to 51.8% (95% CI 48.6%–64.8%). A pronounced clustering of cases was observed in one specific area, whereas herds in the southernmost region exhibited prevalences below 1.5%. Prevalence rates were 10.4% (95% CI 9.9%–13.0%) in lambs and 13.4% (95% CI 12.1%–17.5%) in adult sheep. Additionally, a slight but statistically significant reduction in fat and muscle was associated with acquired melanosis in both age groups.

Analysis of variance revealed no significant differences in element concentrations between livers with and without acquired melanosis, nor were any statistical differences detected in polonium-210 activity concentrations. It should be noted that data on sheep diet were not available; therefore, the results do not account for potential confounding effects associated with the diet, especially during the winter period. The derived annual ^210^Po absorbed doses to livers with and without acquired melanosis were lower than the recommended ERICA whole-body default dose. This study estimated conservative annual average effective doses from ingestion of ^210^Po to children, adults, and individuals most exposed in Greenland. Except for the most exposed children (10th percentile), the doses were not higher than the world average annual effective dose from ingesting naturally occurring radionuclides.

The results indicate no association between the concentrations of ^21^^0^Po, ^21^⁰Pb, Cd, As, F, REE, Zn, U, and Th and the occurrence of acquired melanosis (black discoloration of sheep livers). Based on the measured concentrations of chemical elements and radionuclides, consuming sheep livers from the region does not appear to present a toxicological or radiological risk. Nevertheless, as the biological mechanism underlying acquired melanosis remains unclear, any conclusions regarding the safety of consuming livers affected by melanosis should be drawn cautiously until the aetiology is fully elucidated.

## Supplementary information

Below is the link to the electronic supplementary material.

ESM 1 (XLSX 44.7 KB)

ESM 2 (XLSX 12.6 KB)

## Data Availability

We have uploaded raw data in supporting materials.
